# Expression of genes involved in drug metabolism differs between perfusable 3D liver tissue and conventional 2D‐cultured hepatocellular carcinoma cells

**DOI:** 10.1002/2211-5463.12948

**Published:** 2020-09-15

**Authors:** Nobuhito Mori, Yasuyuki S. Kida

**Affiliations:** ^1^ Cellular and Molecular Biotechnology Research Institute National Institute of Advanced Industrial Science and Technology (AIST) Tsukuba Japan; ^2^ Advanced Photonics and Biosensing Open Innovation Laboratory National Institute of Advanced Industrial Science and Technology (AIST) Tsukuba Japan

**Keywords:** blood vessels, gene expression profile, hepatocellular carcinoma, liver, organoids, RNA‐seq, transcriptome

## Abstract

Tubular 3D liver tissue with enhanced capillary‐like structures branching from a large main channel is potentially useful for drug discovery because the perfusable main channel and capillary‐like structures enable mass transfer into and out from the tissue. Tubular liver tissue is comprised of the hepatocellular carcinoma cell line HepG2, human umbilical vein endothelial cells (HUVECs), and mesenchymal stem cells (MSCs), using a perfusion device functioning as the interface for an external pump. This study aimed to compare the expression of genes involved in drug metabolism between 2D‐cultured hepatocellular carcinoma cells and 3D‐cultured tubular liver tissue. Gene expression profiles of 2D‐cultured cells and tubular liver tissue were compared using RNA sequencing. Multidimensional scaling analysis revealed that culture dimensionality had a more prominent effect on gene expression profiles than perfusion conditions. More specifically, genes involved in drug metabolism such as *CYP2D6*, *CYP2E1*, *NNMT*, and *SLC28A1* were slightly upregulated in the 3D cultures, while certain genes such as *ALDH1B1*, *ALDH1A2*, and *SULT1E1* were downregulated. These results indicate that gene expression profiles are largely influenced by culture dimensionality and are potentially useful to researchers intending to switch from 2D culture to 3D culture of hepatocellular carcinoma or other tissue types.

## Graphical abstract

We compared drug‐metabolizing gene expression between 2D‐cultured hepatocellular carcinoma cells and 3D‐cultured perfusable liver tissue comprising hepatocellular carcinoma cells (HepG2), human umbilical vein endothelial cells, and mesenchymal stem cells by RNA sequencing. The results indicate that gene expression profiles are largely influenced by culture dimensionality and are potentially applicable to researchers intending to switch from 2D culturing to 3D culturing of hepatocellular carcinoma.

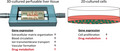

**Abbreviations**
DEG: differentially expressed gene
FC: fold change
FDR: false discovery rate
HUVEC: human umbilical vein endothelial cell
MSC: mesenchymal stem cell

Various 3D liver‐like cultures comprising hepatocellular carcinoma cells, such as spheroids [1, 2], cell‐laden hydrogel [3, 4], and organoids [5, 6], have been developed. Drug discovery studies have increasingly focused on 3D liver‐like tissue cultures using cell culture plates owing to their various advantages over conventional 2D cultures of hepatocellular carcinoma cells, including increased cellular functions such as albumin secretion and xenobiotic metabolism and increased sensitivity to hepatotoxic compounds. To compensate for the lack of a perfusable vascular network in 3D liver‐like tissues, we previously developed a tubular 3D liver‐like culture, called tubular liver tissue, with enhanced capillary‐like structures branching from a large main channel [7], upon combining a perfusion device [8, 9] with a collagen gel populated with the hepatocellular carcinoma cell line HepG2, human umbilical vein endothelial cells (HUVECs), and mesenchymal stem cells (MSCs). The perfusable main channel and capillary‐like structures facilitate the mass transfer of not only oxygen and nutrients but also test substances and their metabolites both into and from the tissue, which can then be sampled, thereby facilitating their applications in drug discovery studies. However, differences in drug‐metabolizing gene expression between tubular liver tissue and the conventional 2D‐cultured HepG2 remain unknown. Herein, we constructed a tubular liver tissue and used RNA sequencing (RNA‐seq) to compare its gene expression profiles under perfused and nonperfused conditions, with that of 2D‐cultured HepG2 cells, HUVECs, and MSCs mixed at the same ratio as that used for generating the liver tissue (Fig. 1). We mainly focused on genes involved in drug metabolism, categorized as phase I, II, III, and nuclear receptor genes. Phase I enzymes are involved in oxidation, reduction, and hydrolysis of xenobiotics. Phase II enzymes are involved in conjugation reactions. Phase III enzymes are usually related to transportation of xenobiotics [10]. Since drug metabolism *in vivo* mainly involves these three phases, research in the field of drug discovery largely targets them. In addition, a set of nuclear receptors are also investigated because it is known that they control hepatic metabolism and hepatotoxicity [11]. Our results will potentially benefit researchers intending to switch from 2D‐cultured hepatocellular carcinoma cells or other 3D liver‐like tissues to tubular liver tissue.

**Fig. 1:**
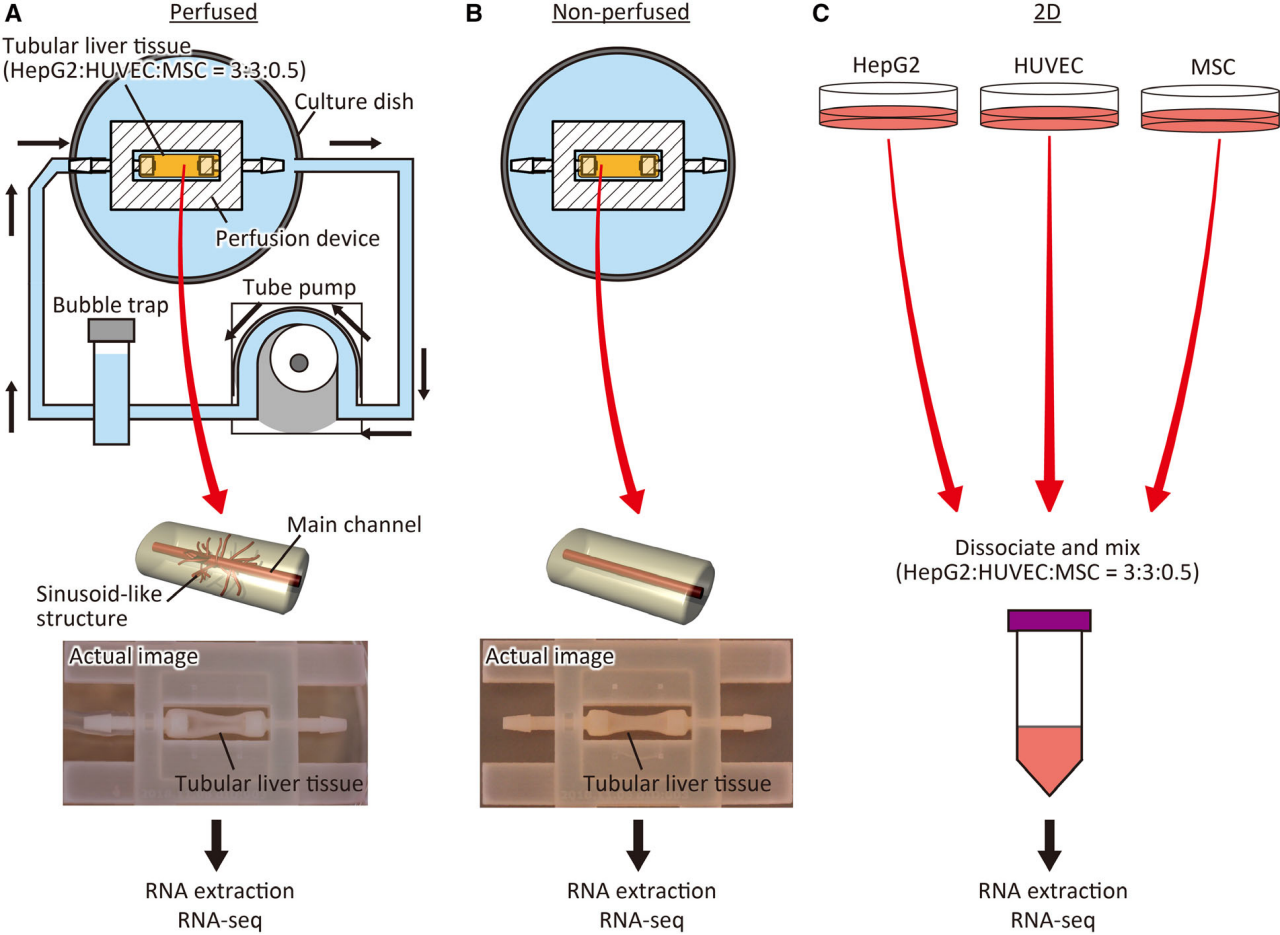
Schematic representation of the experimental design. (A) In the perfused group, the tubular liver tissue was cultured under perfusion with medium, using a tube pump. Total RNA was extracted from tissue detached from the device and used for RNA‐seq. (B) In the nonperfused group, the tubular liver tissue was submerged in the medium and statically cultured. Total RNA was extracted from the tissue detached from the device and subjected to RNA‐seq. (C) In the 2D‐cultured group, hepatocellular carcinoma cell line HepG2, HUVECs, and MSCs were cultured in cell culture dishes. The cells were dissociated, mixed at the same ratio as tubular liver tissues, and used for RNA‐seq after total RNA extraction.

## Materials and methods

### Construction of the tubular liver tissue

Tubular liver tissue was constructed using a perfusion device, as previously described (Fig. 1A,B) [7]. Briefly, the device was filled with a collagen matrix (IAC‐50; Koken Co., Tokyo, Japan) populated with HepG2 cells (3 × 10^7^ cells/mL), HUVECs (3 × 10^7^ cells/mL), and MSCs (0.5 × 10^7^ cells/mL). The ratio of each cell type was determined based on the literature [5, 12]. The needle previously set in the device was extracted. Then, HUVECs were infused into the resultant tunnel to form the main channel. Subsequently, we connected the device to an external pump for perfusion (Fig. 1A) and immersed the device in culture medium for nonperfusion (Fig. 1B). The culture medium was composed of 1 : 1 mix of HepG2 and HUVEC media, which were individually used for the culture of cell populations in 2D conditions.

### 2D cell culture

HepG2 cells, HUVECs, and MSCs were independently cultured for 24 h. HepG2 cells were cultured in cell culture dishes (VTC‐D150; AS ONE Corporation, Osaka, Japan) with low glucose Dulbecco's modified Eagle's medium (DMEM; FUJIFILM Wako Pure Chemical Corporation, Osaka, Japan) containing 10% FBS (Thermo Fisher Scientific Inc., Waltham, MA, USA) and 1% penicillin/streptomycin solution (FUJIFILM Wako Pure Chemical). HUVECs (PromoCell GmbH, Heidelberg, Germany) were cultured in gelatin‐coated cell culture dishes (FUJIFILM Wako Pure Chemical Corporation) containing endothelial cell growth medium 2 (PromoCell) supplemented with 1% penicillin/streptomycin solution (×100). MSCs (SCRC‐4000; ATCC, Manassas, VA, USA) were cultured in gelatin‐coated cell culture dishes containing high‐glucose DMEM (FUJIFILM Wako Pure Chemical Corporation) supplemented with 10% FBS, 1% nonessential amino acids solution (×100) (FUJIFILM Wako Pure Chemical), and 1% penicillin/streptomycin solution (×100). All cells were cultured at 37 °C in a 5% CO_2_ atmosphere. The cells were dissociated with TrypLE Express (Thermo Fisher Scientific) 1 day after plating and mixed at the same ratio as the tubular liver tissue (3 : 3 : 0.5) (Fig. 1C) for extraction of RNA.

### RNA‐seq analysis

Total RNA was extracted from 3D‐ and 2D‐cultured perfused and nonperfused cells using NucleoSpin RNA (Macherey Nagel GmbH & Co. KG, Duren, Germany) and sequenced using NovaSeq 6000 (Illumina Inc., San Diego, CA, USA) with biological duplicates. Read data were processed and analyzed using STAR (2.7.1a) [13], RSEM (1.3.1) [14], and edgeR (3.28.1) [15, 16] with the hg38 reference genome and gene annotation Ensembl GRCh38. The read count data were normalized using the trimmed mean of M values. Gene ontology (GO) enrichment analysis was performed using the “database for annotation, visualisation and integrated discovery” (DAVID) [17, 18] via RDAVIDWebService (1.20.0) [19] and executed on R [20]. The enriched GO terms (adjusted *P*‐value < 0.01) obtained from a category GOTERM_BP_FAT of DAVID were classified into 4 clusters by clustering analysis based on semantic similarity computation [21]. The semantic similarities between all the pairs of the enriched GO terms were calculated using GOSemSim [22]. Then, hierarchical clustering according to the calculated similarities was performed using the hclust function of R.

## Results and Discussion

### Culture dimensionality had a prominent effect on gene expression

**Fig. 2:**
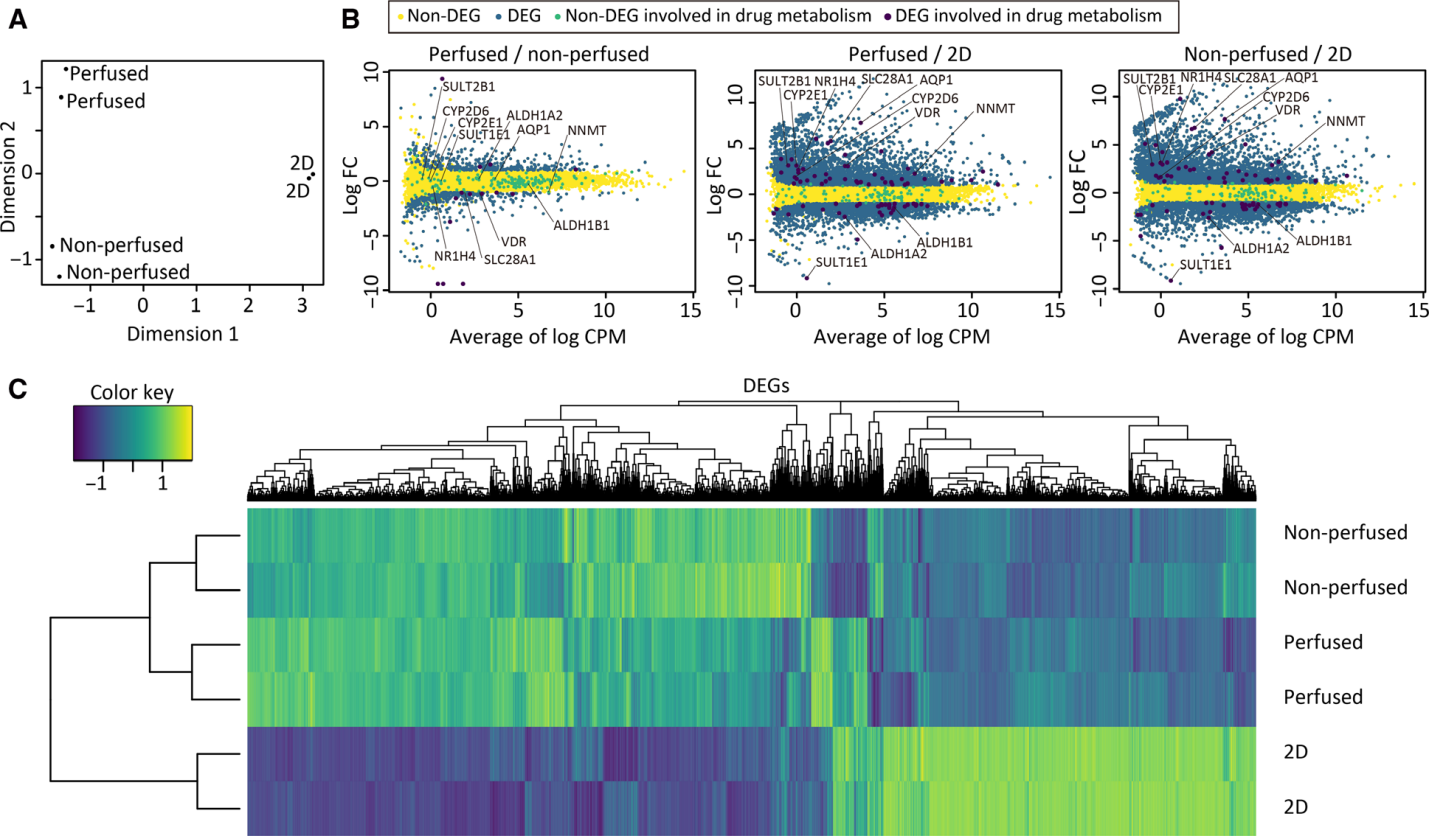
Analysis of gene expression profiles. (A) Multidimensional scaling plot of the perfused and nonperfused samples and 2D‐cultured samples. (B) Scatter plots of log_2_ FC values vs the average log_2_ CPM. CPM, counts per million. DEGs, |log_2 _FC| ≥ 1 and FDR < 0.05. (C) A heat map of the perfused and nonperfused samples, and the 2D‐cultured samples.

To assess overall differences among the three groups, the data were analyzed through multidimensional scaling (Fig. 2A). As expected, data points were clustered in accordance with the culture conditions in a plane of the first two dimensions, indicating that variations of the samples in each group were small enough to evaluate differences among groups and that these conditions had robust effects on gene expression profiles. Thereafter, pairwise group comparisons were carried out (Fig. 2B); 792 (upregulated: 381, downregulated: 411), 5974 (upregulated: 3437, downregulated: 2537), and 6016 (upregulated: 3598, downregulated: 2418) differentially expressed genes [DEGs; |log_2 _FC| ≥ 1 and false discovery rate (FDR) < 0.05] were identified between perfused and nonperfused cells, perfused and 2D‐cultured cells, and nonperfused and 2D‐cultured cells, respectively, suggesting that culture dimensionality (i.e., tubular liver tissue vs 2D) has a more prominent effect on gene expression than perfusion, even though previous studies have reported that perfusion retains tissue viability and functions. This finding was further confirmed through clustering analysis. As shown in the heat map (Fig. 2C), the perfused and nonperfused groups were closely clustered, compared to 2D‐cultured groups. GO enrichment analysis was also performed to investigate the differences between the perfused and 2D‐cultured groups in detail (Tables 1 and 2). The enriched GO terms were classified by clustering analysis based on semantic similarity computation. Consequently, it was revealed that genes involved in extracellular matrix organization, blood circulation, ion transmembrane transport, and vascular formation were enriched in the perfused condition (Table 1), whereas those involved in cell proliferation (Table 2) were enriched in 2D culture condition. This observation indicates that the perfused tubular tissue is physiologically more relevant than the 2D‐cultured cells.

**Table 1:** GO terms (biological process) enriched in the perfused tubular liver tissue compared with the 2D‐cultured cells. GO terms were classified into four clusters by clustering analysis based on semantic similarity computation, and the top three of each cluster are shown

Cluster No.	GO ID	Description	Adjusted *P*‐value
1	GO:0007166	Cell surface receptor signaling pathway	1.63E‐16
	GO:0043062	Extracellular structure organization	8.05E‐14
	GO:0030198	Extracellular matrix organization	8.05E‐14
2	GO:0003008	System process	3.68E‐16
	GO:0009605	Response to external stimulus	3.40E‐16
	GO:0008015	Blood circulation	2.54E‐12
3	GO:0006811	Ion transport	4.55E‐14
	GO:0034220	Ion transmembrane transport	1.53E‐09
	GO:0055085	Transmembrane transport	5.05E‐09
4	GO:0045595	Regulation of cell differentiation	3.49E‐13
	GO:2000026	Regulation of multicellular organismal development	1.13E‐11
	GO:0001944	Vasculature development	6.01E‐09

**Table 2:** GO terms (biological process) enriched in the 2D‐cultured cells compared with the perfused tubular liver tissue. GO terms were classified into four clusters by clustering analysis based on semantic similarity computation, and the top three of each cluster are shown

Cluster No.	GO ID	Description	Adjusted *P*‐value
1	GO:1903047	Mitotic cell cycle process	1.43E‐39
	GO:0000278	Mitotic cell cycle	2.14E‐38
	GO:0022402	Cell cycle process	1.01E‐35
2	GO:0006259	DNA metabolic process	5.12E‐29
	GO:0006260	DNA replication	3.20E‐28
	GO:0034660	ncRNA metabolic process	8.64E‐28
3	GO:0000280	Nuclear division	5.51E‐25
	GO:0051276	Chromosome organization	2.32E‐22
	GO:0048285	Organelle fission	2.37E‐22
4	GO:0032543	Mitochondrial translation	4.37E‐18
	GO:0070125	Mitochondrial translational elongation	5.78E‐15
	GO:0006415	Translational termination	2.84E‐14

### Drug‐metabolizing genes were basically upregulated in the tubular liver tissue

**Fig. 3:**
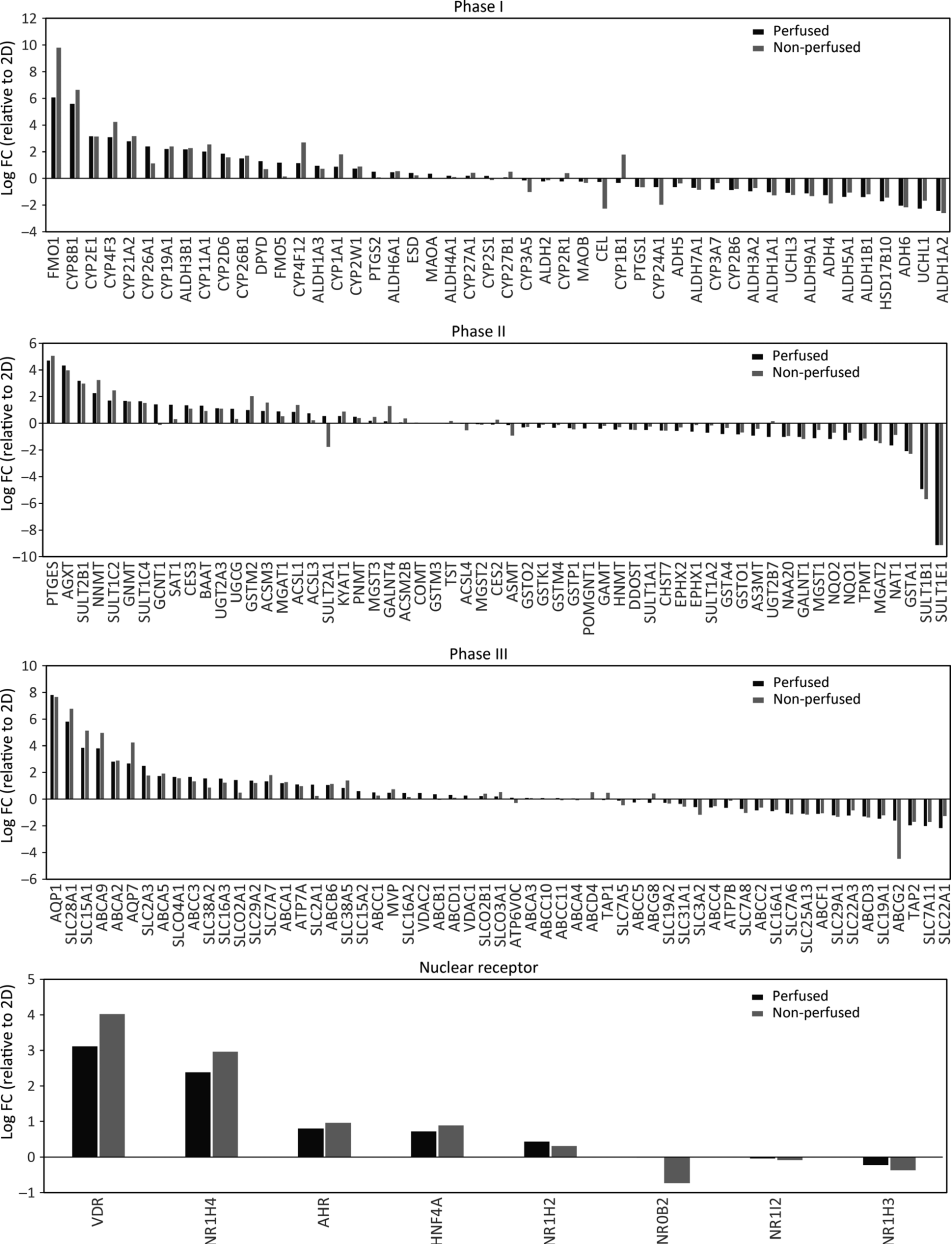
Graphs showing log_2_ FC values of genes associated with drug metabolism. (A) Phase I genes. (B) Phase II genes. (C) Phase III genes. (D) Nuclear receptor genes. The 2D‐cultured group; log_2_ FC = 0.

**Fig. 4:**
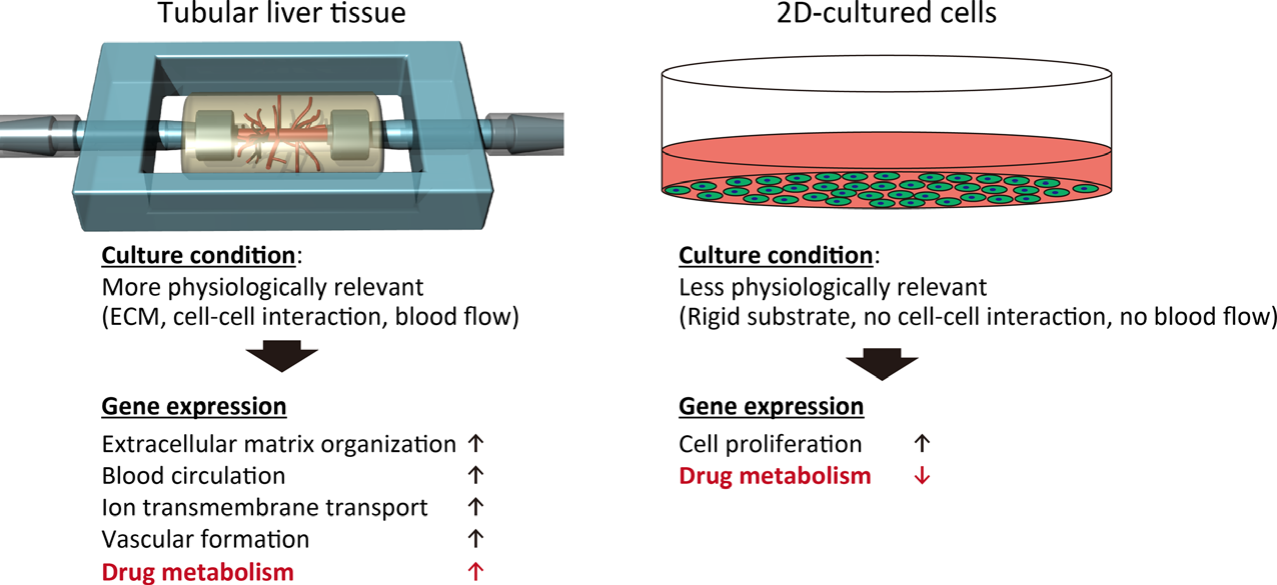
Schematic picture of the comparison between the tubular liver tissue and 2D‐cultured cells.

We evaluated the expression levels of drug‐metabolizing genes classified into phase I, phase II, and phase III, and nuclear receptor genes (Figs 2B and 3, Table 3, 4, 5, 6) as previously described [2, 10, 11]. For the phase I genes, mean log_2_ fold change (FC) values were 0.4 and 0.5 in the perfused and the nonperfused groups (Fig. 3A, Table 3), respectively, indicating that the phase I genes, particularly *CYP2D6* and *CYP2E1,* in both groups were slightly upregulated on an average; *CYP2D6* and *CYP2E1* encoding cytochrome P450 members are involved in metabolism of 20% and 2% of known drugs, respectively [23]. Some phase I genes were downregulated, including aldehyde dehydrogenases such as *ALDH1A2,* which encodes a retinal dehydrogenase with retinaldehyde metabolizing potential but only very low activity with acetaldehyde and propanal, and *ALDH1B1*, which detoxifies alcohol‐derived acetaldehyde [24]. The downregulated genes might be related to cell proliferation since the 2D‐cultured cells were at a highly proliferative state as revealed by GO enrichment analysis (Table 4). For phase II genes, the trend was more neutral; mean log_2_ FC values were 0.0 for both perfused and the nonperfused groups (Fig. 3B, Table 4). These values may have been negatively biased owing to an extremely low log_2_ FC value (−9.1) of *SULT1E1* which encodes a sulfotransferase catalyzing estradiol and estrone sulfation. Among the upregulated phase II genes, prominent drug‐metabolizing genes were identified, including *SULT2B1* which catalyzes pregnenolone and dehydroepiandrosterone sulfation, and *NNMT* which encodes nicotinamide N‐methyltransferase involved in the biotransformation of numerous drugs and xenobiotic compounds [24]. Among phase III genes, mean log_2_ FC values were 0.5 both in perfused and in nonperfused groups (Fig. 3C, Table 5), wherein *SLC28A1* and *AQP1* were particularly upregulated. *SLC28A1* encodes a sodium‐dependent and pyrimidine‐selective transporter involved in uridine, cytidine, thymidine, and nucleoside‐derived drug transport, whereas *AQP1* encodes an aquaporin channel [24]. Among nuclear receptor genes, mean log_2_ FC values were 0.9 and 1.0 in the perfused and nonperfused groups, respectively, indicating higher expression levels than those of phase I–III genes (Fig. 3D, Table 6). In particular, *VDR* encoding the vitamin D receptor and *NR1H4* encoding the bile acid receptor, both associated with hepatotoxicity and metabolism [11], were significantly upregulated. Together, drug‐metabolizing gene expression profiles were similar between perfused and nonperfused groups. Furthermore, these genes were basically upregulated in the tubular liver compared with 2D‐cultured cells, although it should be noted that some genes were downregulated and also that an interaction among different cell types was excluded in the 2D‐culture condition for simplicity. It is assumed that the upregulation of drug‐metabolizing genes was due to the physiologically relevant culture conditions, such as ECM, cell–cell interactions, and blood flow, of the tubular liver tissue (Fig. 4). The metabolism and hepatotoxicity of many drugs, such as midazolam, bufuralol, acetaminophen, and diclofenac, are assessed more accurately by using static 3D culture conditions (spheroid and cell‐laden ECM) rather than 2D conditions [2, 5]. Considering that the perfused tubular liver tissue is more viable (4.6‐fold RNA levels) and more feasible for drug injection into the tissue and metabolite collection than the nonperfused tissue (equivalent to static 3D culture), the perfusable tubular liver tissue might be a promising experimental model for drug discovery studies. Considering the utility of the perfusable tubular liver tissue, the difference with conventional 2D‐cultured cells should be considered.

**Table 3:** Phase I genes. Genes that were not detected by RNA‐seq or omitted due to extremely low counts during edgeR process are shown as blank.

Ensembl ID	Symbol	Description	Log FC			Log CPM	FDR
			Perfused/2D	Nonperfused/2D	Perfused/Nonperfused		
ENSG00000010932	FMO1	Flavin containing monooxygenase 1	6.07	9.79	−3.73	1.10	2.87E‐05
ENSG00000180432	CYP8B1	Cytochrome P450, family 8, subfamily B, polypeptide 1	5.58	6.63	−1.05	1.77	3.59E‐06
ENSG00000130649	CYP2E1	Cytochrome P450, family 2, subfamily E, polypeptide 1	3.15	3.13	0.02	‐0.02	6.09E‐04
ENSG00000186529	CYP4F3	Cytochrome P450, family 4, subfamily F, polypeptide 3	3.08	4.23	−1.15	2.92	4.67E‐06
ENSG00000231852	CYP21A2	Cytochrome P450, family 21, subfamily A, polypeptide 2	2.78	3.16	−0.38	0.36	1.96E‐04
ENSG00000095596	CYP26A1	Cytochrome P450, family 26, subfamily A, polypeptide 1	2.38	1.12	1.26	−0.89	2.14E‐02
ENSG00000137869	CYP19A1	Cytochrome P450, family 19, subfamily A, polypeptide 1	2.20	2.38	−0.18	1.67	3.37E‐05
ENSG00000006534	ALDH3B1	Aldehyde dehydrogenase 3 family, member B1	2.16	2.26	−0.10	4.45	6.43E‐07
ENSG00000140459	CYP11A1	Cytochrome P450, family 11, subfamily A, polypeptide 1	1.99	2.54	−0.55	1.01	2.56E‐04
ENSG00000100197	CYP2D6	Cytochrome P450, family 2, subfamily D, polypeptide 6	1.86	1.57	0.29	−0.12	1.67E‐02
ENSG00000003137	CYP26B1	Cytochrome P450, family 26, subfamily B, polypeptide 1	1.50	1.70	−0.21	0.59	5.72E‐03
ENSG00000188641	DPYD	Dihydropyrimidine dehydrogenase	1.29	0.69	0.60	3.47	2.89E‐04
ENSG00000131781	FMO5	Flavin containing monooxygenase 5	1.18	0.13	1.04	−0.17	3.36E‐02
ENSG00000186204	CYP4F12	Cytochrome P450, family 4, subfamily F, polypeptide 12	1.13	2.70	−1.57	1.43	1.14E‐04
ENSG00000184254	ALDH1A3	Aldehyde dehydrogenase 1 family, member A3	0.93	0.72	0.21	2.62	1.33E‐02
ENSG00000140465	CYP1A1	Cytochrome P450, family 1, subfamily A, polypeptide 1	0.86	1.81	−0.95	0.37	1.90E‐02
ENSG00000073067	CYP2W1	Cytochrome P450, family 2, subfamily W, polypeptide 1	0.74	0.87	−0.14	5.06	4.61E‐05
ENSG00000073756	PTGS2	Prostaglandin‐endoperoxide synthase 2 (prostaglandin G/H synthase and cyclooxygenase)	0.49	0.06	0.43	4.46	1.50E‐02
ENSG00000119711	ALDH6A1	Aldehyde dehydrogenase 6 family, member A1	0.46	0.54	−0.08	3.83	6.91E‐03
ENSG00000139684	ESD	Esterase D	0.40	0.22	0.18	6.43	7.12E‐03
ENSG00000189221	MAOA	Monoamine oxidase A	0.34	0.03	0.31	5.50	1.19E‐02
ENSG00000159423	ALDH4A1	Aldehyde dehydrogenase 4 family, member A1	0.19	0.09	0.10	5.96	2.28E‐01
ENSG00000135929	CYP27A1	Cytochrome P450, family 27, subfamily A, polypeptide 1	0.19	0.42	−0.23	5.10	1.45E‐02
ENSG00000167600	CYP2S1	Cytochrome P450, family 2, subfamily S, polypeptide 1	0.18	−0.13	0.31	4.62	2.59E‐01
ENSG00000111012	CYP27B1	Cytochrome P450, family 27, subfamily B, polypeptide 1	0.07	0.51	−0.44	0.24	4.59E‐01
ENSG00000106258	CYP3A5	Cytochrome P450, family 3, subfamily A, polypeptide 5	−0.16	−1.03	0.87	2.92	6.08E‐04
ENSG00000111275	ALDH2	Aldehyde dehydrogenase 2 family (mitochondrial)	−0.22	−0.14	−0.08	5.98	6.16E‐02
ENSG00000186104	CYP2R1	Cytochrome P450, family 2, subfamily R, polypeptide 1	−0.22	0.39	−0.61	0.43	3.86E‐01
ENSG00000069535	MAOB	Monoamine oxidase B	−0.24	−0.33	0.10	4.23	6.00E‐02
ENSG00000170835	CEL	Carboxyl ester lipase (bile salt‐stimulated lipase)	−0.26	−2.27	2.01	−1.15	3.48E‐02
ENSG00000138061	CYP1B1	Cytochrome P450, family 1, subfamily B, polypeptide 1	−0.32	1.78	−2.10	−0.28	2.64E‐02
ENSG00000095303	PTGS1	Prostaglandin‐endoperoxide synthase 1 (prostaglandin G/H synthase and cyclooxygenase)	−0.64	−0.67	0.03	5.32	4.83E‐04
ENSG00000019186	CYP24A1	Cytochrome P450, family 24, subfamily A, polypeptide 1	−0.65	−1.98	1.33	2.81	3.91E‐05
ENSG00000197894	ADH5	Alcohol dehydrogenase 5 (class III), chi polypeptide	−0.65	−0.37	−0.28	6.86	9.12E‐05
ENSG00000164904	ALDH7A1	Aldehyde dehydrogenase 7 family, member A1	−0.71	−0.86	0.15	5.99	3.09E‐05
ENSG00000160870	CYP3A7	Cytochrome P450, family 3, subfamily A, polypeptide 7	−0.83	−0.34	−0.49	0.12	2.38E‐01
ENSG00000197408	CYP2B6	Cytochrome P450, family 2, subfamily B, polypeptide 6	−0.87	−0.79	−0.08	−0.77	2.42E‐01
ENSG00000072210	ALDH3A2	Aldehyde dehydrogenase 3 family, member A2	−0.97	−0.72	−0.25	5.54	8.87E‐06
ENSG00000165092	ALDH1A1	Aldehyde dehydrogenase 1 family, member A1	−1.04	−1.28	0.24	3.59	6.36E‐05
ENSG00000118939	UCHL3	Ubiquitin carboxyl‐terminal esterase L3 (ubiquitin thiolesterase)	−1.08	−1.25	0.17	4.84	8.72E‐07
ENSG00000143149	ALDH9A1	Aldehyde dehydrogenase 9 family, member A1	−1.12	−1.31	0.19	5.06	8.58E‐07
ENSG00000198099	ADH4	Alcohol dehydrogenase 4 (class II), pi polypeptide	−1.27	−1.88	0.62	0.35	3.45E‐03
ENSG00000112294	ALDH5A1	Aldehyde dehydrogenase 5 family, member A1	−1.38	−1.07	−0.31	5.28	5.88E‐07
ENSG00000137124	ALDH1B1	Aldehyde dehydrogenase 1 family, member B1	−1.39	−1.19	−0.20	5.56	4.83E‐07
ENSG00000072506	HSD17B10	Hydroxysteroid (17‐beta) dehydrogenase 10	−1.71	−1.44	−0.27	5.50	7.48E‐08
ENSG00000172955	ADH6	Alcohol dehydrogenase 6 (class V)	−2.04	−2.18	0.14	2.41	2.59E‐05
ENSG00000154277	UCHL1	Ubiquitin carboxyl‐terminal esterase L1 (ubiquitin thiolesterase)	−2.28	−1.68	−0.60	4.86	5.23E‐07
ENSG00000128918	ALDH1A2	Aldehyde dehydrogenase 1 family, member A2	−2.43	−2.60	0.16	2.74	5.64E‐06
ENSG00000114771	AADAC	Arylacetamide deacetylase (esterase)					
ENSG00000187758	ADH1A	Alcohol dehydrogenase 1A (class I), alpha polypeptide					
ENSG00000196616	ADH1B	Alcohol dehydrogenase 1B (class I), beta polypeptide					
ENSG00000248144	ADH1C	Alcohol dehydrogenase 1C (class I), gamma polypeptide					
ENSG00000196344	ADH7	Alcohol dehydrogenase 7 (class IV), mu or sigma polypeptide					
ENSG00000108602	ALDH3A1	Aldehyde dehydrogenase 3 family, member A1					
ENSG00000132746	ALDH3B2	Aldehyde dehydrogenase 3 family, member B2					
ENSG00000118514	ALDH8A1	Aldehyde dehydrogenase 8 family, member A1					
ENSG00000160882	CYP11B1	Cytochrome P450, family 11, subfamily B, polypeptide 1					
ENSG00000179142	CYP11B2	Cytochrome P450, family 11, subfamily B, polypeptide 2					
ENSG00000148795	CYP17A1	Cytochrome P450, family 17, subfamily A, polypeptide 1					
ENSG00000140505	CYP1A2	Cytochrome P450, family 1, subfamily A, polypeptide 2					
ENSG00000187553	CYP26C1	Cytochrome P450, family 26, subfamily C, polypeptide 1					
ENSG00000197838	CYP2A13	Cytochrome P450, family 2, subfamily A, polypeptide 13					
ENSG00000108242	CYP2C18	Cytochrome P450, family 2, subfamily C, polypeptide 18					
ENSG00000165841	CYP2C19	Cytochrome P450, family 2, subfamily C, polypeptide 19					
ENSG00000138115	CYP2C8	Cytochrome P450, family 2, subfamily C, polypeptide 8					
ENSG00000138109	CYP2C9	Cytochrome P450, family 2, subfamily C, polypeptide 9					
ENSG00000197446	CYP2F1	Cytochrome P450, family 2, subfamily F, polypeptide 1					
ENSG00000160868	CYP3A4	Cytochrome P450, family 3, subfamily A, polypeptide 4					
ENSG00000021461	CYP3A43	Cytochrome P450, family 3, subfamily A, polypeptide 43					
ENSG00000187048	CYP4A11	Cytochrome P450, family 4, subfamily A, polypeptide 11					
ENSG00000162365	CYP4A22	Cytochrome P450, family 4, subfamily A, polypeptide 22					
ENSG00000142973	CYP4B1	Cytochrome P450, family 4, subfamily B, polypeptide 1					
ENSG00000171903	CYP4F11	Cytochrome P450, family 4, subfamily F, polypeptide 11					
ENSG00000186115	CYP4F2	Cytochrome P450, family 4, subfamily F, polypeptide 2					
ENSG00000186526	CYP4F8	Cytochrome P450, family 4, subfamily F, polypeptide 8					
ENSG00000167910	CYP7A1	Cytochrome P450, family 7, subfamily A, polypeptide 1					
ENSG00000172817	CYP7B1	Cytochrome P450, family 7, subfamily B, polypeptide 1					
ENSG00000100867	DHRS2	Dehydrogenase/reductase (SDR family) member 2					
ENSG00000094963	FMO2	Flavin containing monooxygenase 2 (nonfunctional)					
ENSG00000007933	FMO3	Flavin containing monooxygenase 3					
ENSG00000076258	FMO4	Flavin containing monooxygenase 4					
ENSG00000145649	GZMA	Granzyme A (granzyme 1, cytotoxic T lymphocyte‐associated serine esterase 3)					
ENSG00000100453	GZMB	Granzyme B (granzyme 2, cytotoxic T lymphocyte‐associated serine esterase 1)					
ENSG00000158125	XDH	Xanthine dehydrogenase					

**Table 4:** Phase II genes. Genes that were not detected by RNA‐seq or omitted due to extremely low counts during edgeR process are shown as blank.

EnsemblID	Symbol	Description	Log FC			Log CPM	FDR
			Perfused/2D	Nonperfused/2D	Perfused/Nonperfused		
ENSG00000148344	PTGES	Prostaglandin E synthase	4.70	5.06	−0.37	4.83	2.99E‐07
ENSG00000172482	AGXT	Alanine‐glyoxylate aminotransferase	4.32	3.98	0.34	2.76	2.16E‐06
ENSG00000088002	SULT2B1	Sulfotransferase family, cytosolic, 2B, member 1	3.17	2.97	0.20	−0.49	1.27E‐03
ENSG00000166741	NNMT	Nicotinamide N‐methyltransferase	2.25	3.23	−0.98	6.73	5.00E‐08
ENSG00000198203	SULT1C2	Sulfotransferase family, cytosolic, 1C, member 2	1.71	2.47	−0.76	1.88	4.77E‐05
ENSG00000124713	GNMT	Glycine‐N‐methyltransferase	1.67	1.63	0.04	−0.01	1.08E‐02
ENSG00000198075	SULT1C4	Sulfotransferase family, cytosolic, 1C, member 4	1.65	1.53	0.12	2.75	9.40E‐05
ENSG00000187210	GCNT1	Glucosaminyl (N‐acetyl) transferase 1, core 2	1.42	−0.13	1.55	3.40	8.53E‐05
ENSG00000130066	SAT1	Spermidine/spermine N1‐acetyltransferase 1	1.39	0.30	1.08	8.29	1.11E‐05
ENSG00000172828	CES3	Carboxylesterase 3	1.35	1.10	0.25	3.66	4.72E‐05
ENSG00000136881	BAAT	Bile acid CoA: amino acid N‐acyltransferase (glycine‐N‐choloyltransferase)	1.31	0.91	0.41	5.05	4.02E‐05
ENSG00000135220	UGT2A3	UDP glucuronosyltransferase 2 family, polypeptide A3	1.12	1.09	0.02	0.79	8.30E‐02
ENSG00000148154	UGCG	UDP/glucose ceramide glucosyltransferase	1.08	0.33	0.76	5.45	3.57E‐06
ENSG00000213366	GSTM2	Glutathione S‐transferase mu 2 (muscle)	0.99	2.03	−1.04	3.76	6.29E‐07
ENSG00000005187	ACSM3	Acyl‐CoA synthetase medium‐chain family member 3	0.91	1.55	−0.64	3.61	4.27E‐05
ENSG00000131446	MGAT1	Mannosyl (alpha‐1,3‐)‐glycoprotein beta‐1,2‐N‐acetylglucosaminyltransferase	0.89	0.54	0.35	7.30	8.62E‐05
ENSG00000151726	ACSL1	Acyl‐CoA synthetase long‐chain family member 1	0.85	1.37	−0.53	6.10	1.08E‐06
ENSG00000123983	ACSL3	Acyl‐CoA synthetase long‐chain family member 3	0.76	0.23	0.53	6.27	1.10E‐05
ENSG00000105398	SULT2A1	Sulfotransferase family, cytosolic, 2A, dehydroepiandrosterone (DHEA)‐preferring, member 1	0.54	−1.76	2.31	−0.81	9.08E‐02
ENSG00000141744	PNMT	Phenylethanolamine N‐methyltransferase	0.48	0.39	0.09	0.42	4.40E‐01
ENSG00000143198	MGST3	Microsomal glutathione S‐transferase 3	0.18	0.48	−0.29	5.43	1.42E‐03
ENSG00000257594	GALNT4	UDP‐N‐acetyl‐alpha‐D‐galactosamine:polypeptide N‐acetylgalactosaminyltransferase 4 (GalNAc‐T4)	0.13	1.29	−1.16	4.68	1.72E‐04
ENSG00000066813	ACSM2B	Acyl‐CoA synthetase medium‐chain family member 2B	0.06	0.36	−0.31	−1.06	8.29E‐01
ENSG00000093010	COMT	Catechol‐O‐methyltransferase	0.04	0.03	0.02	6.52	8.30E‐01
ENSG00000134202	GSTM3	Glutathione S‐transferase mu 3 (brain)	−0.02	0.03	−0.05	5.25	8.42E‐01
ENSG00000128311	TST	Thiosulfate sulfurtransferase (rhodanese)	−0.03	0.17	−0.20	4.75	1.47E‐01
ENSG00000068366	ACSL4	Acyl‐CoA synthetase long‐chain family member 4	−0.04	−0.51	0.47	7.15	1.03E‐04
ENSG00000085871	MGST2	Microsomal glutathione S‐transferase 2	−0.07	−0.12	0.05	5.31	5.11E‐01
ENSG00000172831	CES2	Carboxylesterase 2	−0.10	0.27	−0.37	4.85	3.06E‐02
ENSG00000196433	ASMT	Acetylserotonin O‐methyltransferase	−0.14	−0.93	0.79	−0.61	3.20E‐01
ENSG00000065621	GSTO2	Glutathione S‐transferase omega 2	−0.31	−0.29	−0.02	4.28	3.15E‐02
ENSG00000197448	GSTK1	Glutathione S‐transferase kappa 1	−0.33	−0.11	−0.21	5.83	6.86E‐03
ENSG00000168765	GSTM4	Glutathione S‐transferase mu 4	−0.33	−0.14	−0.19	3.96	1.21E‐01
ENSG00000084207	GSTP1	Glutathione S‐transferase pi 1	−0.35	−0.45	0.09	6.00	5.48E‐03
ENSG00000085998	POMGNT1	Protein O‐linked mannose beta1,2‐N‐acetylglucosaminyltransferase	−0.38	−0.01	−0.37	5.86	1.85E‐03
ENSG00000130005	GAMT	Guanidinoacetate N‐methyltransferase	−0.39	−0.20	−0.19	5.00	1.27E‐02
ENSG00000150540	HNMT	Histamine N‐methyltransferase	−0.46	−0.30	−0.17	5.56	2.70E‐02
ENSG00000244038	DDOST	Dolichyl‐diphosphooligosaccharide‐‐protein glycosyltransferase	−0.47	−0.49	0.03	9.26	2.97E‐04
ENSG00000196502	SULT1A1	Sulfotransferase family, cytosolic, 1A, phenol‐preferring, member 1	−0.49	−0.26	−0.23	5.97	1.74E‐02
ENSG00000147119	CHST7	Carbohydrate (N‐acetylglucosamine 6‐O) sulfotransferase 7	−0.54	−0.55	0.01	1.64	1.57E‐01
ENSG00000120915	EPHX2	Epoxide hydrolase 2, cytoplasmic	−0.56	−0.31	−0.25	3.49	4.53E‐02
ENSG00000143819	EPHX1	Epoxide hydrolase 1, microsomal (xenobiotic)	−0.62	−0.14	−0.47	5.03	1.12E‐03
ENSG00000197165	SULT1A2	Sulfotransferase family, cytosolic, 1A, phenol‐preferring, member 2	−0.71	−0.17	−0.53	3.00	7.07E‐02
ENSG00000170899	GSTA4	Glutathione S‐transferase alpha 4	−0.81	−0.33	−0.47	4.42	6.07E‐04
ENSG00000148834	GSTO1	Glutathione S‐transferase omega 1	−0.83	−0.69	−0.14	6.90	3.80E‐06
ENSG00000214435	AS3MT	Arsenic (+3 oxidation state) methyltransferase	−0.92	−0.40	−0.52	3.95	1.33E‐03
ENSG00000171234	UGT2B7	UDP glucuronosyltransferase 2 family, polypeptide B7	−1.02	0.15	−1.17	2.23	2.21E‐03
ENSG00000173418	NAA20	N(alpha)‐acetyltransferase 20, NatB catalytic subunit	−1.02	−0.96	−0.07	5.59	3.12E‐06
ENSG00000141429	GALNT1	UDP‐N‐acetyl‐alpha‐D‐galactosamine:polypeptide N‐acetylgalactosaminyltransferase 1 (GalNAc‐T1)	−1.03	−1.17	0.14	6.68	5.70E‐07
ENSG00000008394	MGST1	Microsomal glutathione S‐transferase 1	−1.13	−0.49	−0.64	6.33	1.33E‐06
ENSG00000124588	NQO2	NAD(P)H dehydrogenase, quinone 2	−1.17	−0.70	−0.47	4.87	3.92E‐06
ENSG00000181019	NQO1	NAD(P)H dehydrogenase, quinone 1	−1.27	−0.70	−0.56	7.46	2.89E‐05
ENSG00000137364	TPMT	Thiopurine S‐methyltransferase	−1.28	−1.14	−0.14	4.65	3.83E‐06
ENSG00000168282	MGAT2	Mannosyl (alpha‐1,6‐)‐glycoprotein beta‐1,2‐N‐acetylglucosaminyltransferase	−1.31	−1.49	0.18	4.68	8.21E‐07
ENSG00000171428	NAT1	N‐acetyltransferase 1 (arylamine N‐acetyltransferase)	−1.65	−0.87	−0.78	1.49	8.77E‐03
ENSG00000243955	GSTA1	Glutathione S‐transferase alpha 1	−2.08	−2.29	0.21	−1.31	1.19E‐02
ENSG00000173597	SULT1B1	Sulfotransferase family, cytosolic, 1B, member 1	−4.93	−5.69	0.76	3.47	1.05E‐08
ENSG00000109193	SULT1E1	Sulfotransferase family 1E, estrogen‐preferring, member 1	−9.13	−9.13	0.00	0.57	2.27E‐05
ENSG00000129673	AANAT	Aralkylamine N‐acetyltransferase					
ENSG00000166743	ACSM1	Acyl‐CoA synthetase medium‐chain family member 1					
ENSG00000171097	CCBL1	Cysteine conjugate‐beta lyase, cytoplasmic					
ENSG00000198848	CES1	Carboxylesterase 1					
ENSG00000159398	CES5A	Carboxylesterase 5A					
ENSG00000149124	GLYAT	Glycine‐N‐acyltransferase					
ENSG00000174156	GSTA3	Glutathione S‐transferase alpha 3					
ENSG00000182793	GSTA5	Glutathione S‐transferase alpha 5					
ENSG00000134201	GSTM5	Glutathione S‐transferase mu 5					
ENSG00000277656	GSTT1	Glutathione S‐transferase theta 1					
ENSG00000241644	INMT	Indolethylamine N‐methyltransferase					
ENSG00000156006	NAT2	N‐acetyltransferase 2 (arylamine N‐acetyltransferase)					
ENSG00000196228	SULT1C3	Sulfotransferase family, cytosolic, 1C, member 3					
ENSG00000130540	SULT4A1	Sulfotransferase family 4A, member 1					
ENSG00000138068	SULT6B1	Sulfotransferase family, cytosolic, 6B, member 1					
ENSG00000241635	UGT1A1	UDP glucuronosyltransferase 1 family, polypeptide A1					
ENSG00000244474	UGT1A4	UDP glucuronosyltransferase 1 family, polypeptide A4					
ENSG00000241119	UGT1A9	UDP glucuronosyltransferase 1 family, polypeptide A9					
ENSG00000173610	UGT2A1	UDP glucuronosyltransferase 2 family, polypeptide A1, complex locus					
ENSG00000109181	UGT2B10	UDP glucuronosyltransferase 2 family, polypeptide B10					
ENSG00000197888	UGT2B17	UDP glucuronosyltransferase 2 family, polypeptide B17					
ENSG00000135226	UGT2B28	UDP glucuronosyltransferase 2 family, polypeptide B28					
ENSG00000156096	UGT2B4	UDP glucuronosyltransferase 2 family, polypeptide B4					
ENSG00000145626	UGT3A1	UDP glycosyltransferase 3 family, polypeptide A1					
ENSG00000174607	UGT8	UDP glycosyltransferase 8					

**Table 5:** Phase III genes. Genes that were not detected by RNA‐seq or omitted due to extremely low counts during edgeR process are shown as blank.

EnsemblID	Symbol	Description	Log FC			Log CPM	FDR
			Perfused/2D	Nonperfused/2D	Perfused/Nonperfused		
ENSG00000240583	AQP1	Aquaporin 1 (Colton blood group)	7.81	7.65	0.16	3.65	1.45E‐06
ENSG00000156222	SLC28A1	Solute carrier family 28 (sodium‐coupled nucleoside transporter), member 1	5.80	6.77	−0.96	1.93	2.42E‐06
ENSG00000088386	SLC15A1	Solute carrier family 15 (oligopeptide transporter), member 1	3.84	5.12	−1.28	−0.90	4.68E‐04
ENSG00000154258	ABCA9	ATP‐binding cassette, subfamily A (ABC1), member 9	3.81	4.96	−1.15	−0.30	7.03E‐04
ENSG00000107331	ABCA2	ATP‐binding cassette, subfamily A (ABC1), member 2	2.81	2.89	−0.08	6.35	2.02E‐08
ENSG00000165269	AQP7	Aquaporin 7	2.67	4.24	−1.57	0.08	9.65E‐04
ENSG00000059804	SLC2A3	Solute carrier family 2 (facilitated glucose transporter), member 3	2.49	1.77	0.72	10.64	6.39E‐08
ENSG00000154265	ABCA5	ATP‐binding cassette, subfamily A (ABC1), member 5	1.74	1.89	−0.16	4.89	1.71E‐06
ENSG00000101187	SLCO4A1	Solute carrier organic anion transporter family, member 4A1	1.67	1.57	0.10	7.31	9.85E‐06
ENSG00000108846	ABCC3	ATP‐binding cassette, subfamily C (CFTR/MRP), member 3	1.66	1.32	0.33	6.00	8.97E‐07
ENSG00000134294	SLC38A2	Solute carrier family 38, member 2	1.54	0.86	0.69	9.97	3.94E‐06
ENSG00000141526	SLC16A3	Solute carrier family 16, member 3 (monocarboxylic acid transporter 4)	1.53	1.23	0.30	8.84	1.53E‐07
ENSG00000174640	SLCO2A1	Solute carrier organic anion transporter family, member 2A1	1.43	0.48	0.95	1.65	9.85E‐04
ENSG00000174669	SLC29A2	Solute carrier family 29 (nucleoside transporters), member 2	1.39	1.20	0.18	0.20	2.40E‐02
ENSG00000155465	SLC7A7	Solute carrier family 7 (amino acid transporter light chain, y + L system), member 7	1.32	1.81	−0.48	5.40	8.39E‐07
ENSG00000165029	ABCA1	ATP‐binding cassette, subfamily A (ABC1), member 1	1.18	1.26	−0.08	6.33	9.56E‐05
ENSG00000165240	ATP7A	ATPase, Cu++ transporting, alpha polypeptide	1.08	0.96	0.12	4.11	1.38E‐04
ENSG00000117394	SLC2A1	Solute carrier family 2 (facilitated glucose transporter), member 1	1.08	0.24	0.84	11.49	1.88E‐05
ENSG00000115657	ABCB6	ATP‐binding cassette, subfamily B (MDR/TAP), member 6	1.05	1.13	−0.08	6.17	1.07E‐06
ENSG00000017483	SLC38A5	Solute carrier family 38, member 5	0.82	1.40	−0.58	6.66	4.93E‐07
ENSG00000163406	SLC15A2	Solute carrier family 15 (H+/peptide transporter), member 2	0.61	0.00	0.61	−0.45	3.70E‐01
ENSG00000103222	ABCC1	ATP‐binding cassette, subfamily C (CFTR/MRP), member 1	0.50	0.26	0.24	7.39	3.48E‐04
ENSG00000013364	MVP	Major vault protein	0.47	0.73	−0.27	7.53	1.23E‐04
ENSG00000147100	SLC16A2	Solute carrier family 16, member 2 (monocarboxylic acid transporter 8)	0.46	0.15	0.31	2.16	2.28E‐01
ENSG00000165637	VDAC2	Voltage‐dependent anion channel 2	0.46	−0.01	0.46	8.19	1.51E‐03
ENSG00000085563	ABCB1	ATP‐binding cassette, subfamily B (MDR/TAP), member 1	0.36	−0.06	0.43	1.56	2.83E‐01
ENSG00000101986	ABCD1	ATP‐binding cassette, subfamily D (ALD), member 1	0.31	0.09	0.22	5.17	1.33E‐02
ENSG00000213585	VDAC1	Voltage‐dependent anion channel 1	0.26	0.03	0.23	8.31	1.24E‐02
ENSG00000137491	SLCO2B1	Solute carrier organic anion transporter family, member 2B1	0.22	0.40	−0.19	4.61	2.23E‐02
ENSG00000176463	SLCO3A1	Solute carrier organic anion transporter family, member 3A1	0.19	0.53	−0.33	0.28	4.53E‐01
ENSG00000185883	ATP6V0C	ATPase, H + transporting, lysosomal 16 kDa, V0 subunit c	0.09	−0.30	0.39	6.34	3.62E‐03
ENSG00000167972	ABCA3	ATP‐binding cassette, subfamily A (ABC1), member 3	0.08	0.06	0.02	4.99	6.69E‐01
ENSG00000124574	ABCC10	ATP‐binding cassette, subfamily C (CFTR/MRP), member 10	0.06	−0.02	0.08	5.09	6.46E‐01
ENSG00000121270	ABCC11	ATP‐binding cassette, subfamily C (CFTR/MRP), member 11	0.06	−0.09	0.15	1.53	8.47E‐01
ENSG00000198691	ABCA4	ATP‐binding cassette, subfamily A (ABC1), member 4	0.03	−0.09	0.12	1.66	9.42E‐01
ENSG00000119688	ABCD4	ATP‐binding cassette, subfamily D (ALD), member 4	0.00	0.52	−0.52	5.08	4.08E‐04
ENSG00000168394	TAP1	Transporter 1, ATP‐binding cassette, subfamily B (MDR/TAP)	−0.07	0.47	−0.54	4.18	5.41E‐03
ENSG00000103257	SLC7A5	Solute carrier family 7 (amino acid transporter light chain, L system), member 5	−0.12	−0.45	0.33	8.22	1.66E‐03
ENSG00000114770	ABCC5	ATP‐binding cassette, subfamily C (CFTR/MRP), member 5	−0.25	−0.06	−0.19	5.58	1.61E‐01
ENSG00000143921	ABCG8	ATP‐binding cassette, subfamily G (WHITE), member 8	−0.29	0.41	−0.70	0.50	2.40E‐01
ENSG00000117479	SLC19A2	Solute carrier family 19 (thiamine transporter), member 2	−0.29	−0.34	0.05	4.68	2.23E‐02
ENSG00000136868	SLC31A1	Solute carrier family 31 (copper transporters), member 1	−0.35	−0.56	0.21	5.77	1.84E‐03
ENSG00000168003	SLC3A2	Solute carrier family 3 (activators of dibasic and neutral amino acid transport), member 2	−0.59	−1.18	0.58	7.14	3.69E‐06
ENSG00000125257	ABCC4	ATP‐binding cassette, subfamily C (CFTR/MRP), member 4	−0.62	−0.52	−0.10	5.11	3.20E‐04
ENSG00000123191	ATP7B	ATPase, Cu++ transporting, beta polypeptide	−0.65	−0.13	−0.52	4.75	2.45E‐03
ENSG00000092068	SLC7A8	Solute carrier family 7 (amino acid transporter light chain, L system), member 8	−0.73	−1.03	0.30	2.59	9.36E‐04
ENSG00000023839	ABCC2	ATP‐binding cassette, subfamily C (CFTR/MRP), member 2	−0.84	−0.63	−0.22	8.62	1.38E‐04
ENSG00000155380	SLC16A1	Solute carrier family 16, member 1 (monocarboxylic acid transporter 1)	−0.90	−0.80	−0.11	6.92	5.09E‐06
ENSG00000103064	SLC7A6	Solute carrier family 7 (amino acid transporter light chain, y + L system), member 6	−1.07	−1.15	0.08	7.12	6.46E‐07
ENSG00000004864	SLC25A13	Solute carrier family 25, member 13 (citrin)	−1.10	−1.16	0.06	5.29	1.26E‐06
ENSG00000204574	ABCF1	ATP‐binding cassette, subfamily F (GCN20), member 1	−1.11	−1.07	−0.04	6.83	3.66E‐07
ENSG00000112759	SLC29A1	Solute carrier family 29 (nucleoside transporters), member 1	−1.23	−1.33	0.11	6.80	2.45E‐07
ENSG00000146477	SLC22A3	Solute carrier family 22 (extraneuronal monoamine transporter), member 3	−1.24	−0.85	−0.39	3.45	8.53E‐05
ENSG00000117528	ABCD3	ATP‐binding cassette, subfamily D (ALD), member 3	−1.30	−1.38	0.08	6.17	2.17E‐07
ENSG00000173638	SLC19A1	Solute carrier family 19 (folate transporter), member 1	−1.46	−1.23	−0.23	5.28	8.00E‐07
ENSG00000118777	ABCG2	ATP‐binding cassette, subfamily G (WHITE), member 2	−1.60	−4.49	2.89	−1.16	1.65E‐03
ENSG00000204267	TAP2	Transporter 2, ATP‐binding cassette, subfamily B (MDR/TAP)	−1.96	−1.70	−0.27	4.74	1.17E‐06
ENSG00000151012	SLC7A11	Solute carrier family 7 (anionic amino acid transporter light chain, xc‐ system), member 11	−2.02	−1.71	−0.31	5.39	1.04E‐05
ENSG00000175003	SLC22A1	Solute carrier family 22 (organic cation transporter), member 1	−2.17	−1.28	−0.89	−0.48	8.09E‐03
ENSG00000144452	ABCA12	ATP‐binding cassette, subfamily A (ABC1), member 12					
ENSG00000179869	ABCA13	ATP‐binding cassette, subfamily A (ABC1), member 13					
ENSG00000073734	ABCB11	ATP‐binding cassette, subfamily B (MDR/TAP), member 11					
ENSG00000005471	ABCB4	ATP‐binding cassette, subfamily B (MDR/TAP), member 4					
ENSG00000004846	ABCB5	ATP‐binding cassette, subfamily B (MDR/TAP), member 5					
ENSG00000140798	ABCC12	ATP‐binding cassette, subfamily C (CFTR/MRP), member 12					
ENSG00000103569	AQP9	Aquaporin 9					
ENSG00000100652	SLC10A1	Solute carrier family 10 (sodium/bile acid cotransporter family), member 1					
ENSG00000125255	SLC10A2	Solute carrier family 10 (sodium/bile acid cotransporter family), member 2					
ENSG00000135917	SLC19A3	Solute carrier family 19, member 3					
ENSG00000112499	SLC22A2	Solute carrier family 22 (organic cation transporter), member 2					
ENSG00000197901	SLC22A6	Solute carrier family 22 (organic anion transporter), member 6					
ENSG00000137204	SLC22A7	Solute carrier family 22 (organic anion transporter), member 7					
ENSG00000149452	SLC22A8	Solute carrier family 22 (organic anion transporter), member 8					
ENSG00000149742	SLC22A9	Solute carrier family 22 (organic anion transporter), member 9					
ENSG00000137860	SLC28A2	Solute carrier family 28 (sodium‐coupled nucleoside transporter), member 2					
ENSG00000197506	SLC28A3	Solute carrier family 28 (sodium‐coupled nucleoside transporter), member 3					
ENSG00000163581	SLC2A2	Solute carrier family 2 (facilitated glucose transporter), member 2					
ENSG00000138079	SLC3A1	Solute carrier family 3 (cystine, dibasic and neutral amino acid transporters, activator of cystine, dibasic and neutral amino acid transport), member 1					
ENSG00000100170	SLC5A1	Solute carrier family 5 (sodium/glucose cotransporter), member 1					
ENSG00000100191	SLC5A4	Solute carrier family 5 (low affinity glucose cotransporter), member 4					
ENSG00000021488	SLC7A9	Solute carrier family 7 (glycoprotein‐associated amino acid transporter light chain, bo,+ system), member 9					
ENSG00000084453	SLCO1A2	Solute carrier organic anion transporter family, member 1A2					
ENSG00000134538	SLCO1B1	Solute carrier organic anion transporter family, member 1B1					
ENSG00000111700	SLCO1B3	Solute carrier organic anion transporter family, member 1B3					

**Table 6:** Nuclear receptor genes. Genes that were not detected by RNA‐seq or omitted due to extremely low counts during edgeR process are shown as blank.

EnsemblID	Symbol	Description	Log FC			Log CPM	FDR
			Perfused/2D	Nonperfused/2D	Perfused/Nonperfused		
ENSG00000111424	VDR	Vitamin D receptor	3.11	4.02	−0.91	2.76	3.96E‐06
ENSG00000012504	NR1H4	Farnesoid X receptor; nuclear receptor subfamily 1 group H member 4	2.38	2.96	−0.58	0.09	2.14E‐03
ENSG00000106546	AHR	Aryl hydrocarbon receptor	0.80	0.97	−0.17	4.06	5.54E‐04
ENSG00000101076	HNF4A	Hepatocyte nuclear factor 4 alpha	0.72	0.89	−0.18	6.26	1.32E‐05
ENSG00000131408	NR1H2	Liver X receptor beta; nuclear receptor subfamily 1 group H member 2	0.43	0.31	0.12	5.01	2.81E‐03
ENSG00000131910	NR0B2	Small heterodimer partner; nuclear receptor subfamily 0 group B member 2	−0.01	−0.73	0.72	4.34	4.39E‐04
ENSG00000144852	NR1I2	Pregnane X receptor; nuclear receptor subfamily 1 group I member 2	−0.04	−0.08	0.05	3.48	8.72E‐01
ENSG00000025434	NR1H3	Liver X receptor alpha; nuclear receptor subfamily 1 group H member 3	−0.22	−0.37	0.14	4.62	1.28E‐02
ENSG00000143257	NR1I3	Constitutive androstane receptor; nuclear receptor subfamily 1 group I member 3					

## Conclusions

In conclusion, herein we compared the gene expression profiles of three groups: perfused and nonperfused tubular liver tissues and conventional 2D‐cultured hepatocellular carcinoma cells. Although all three groups displayed adequate differences among one another to be clearly clustered, differences resulting from culture dimensionality (i.e., tubular liver tissue or 2D‐cultured cells) were particularly significant. Furthermore, assessment of drug‐metabolizing gene expression revealed that expression patterns differed between tubular liver tissues and 2D‐cultured cells, being upregulated in the tubular liver tissue on average. The present results are potentially relevant to researchers using perfusable tubular liver tissues.

## Conflict of interest

The authors declare no conflict of interest.

## Author contributions

NM and YSK conceived the study design, analyzed the data, and wrote the manuscript. YSK supervised the experimental design.

## Data Availability

RNA‐seq data are available in the DNA Data Bank of Japan Sequence Read Archive under accession number DRA008972 and DRA010163 for the tubular liver tissues and 2D‐cultured cells, respectively. The raw data are available from the corresponding author upon reasonable request.
